# Optimization of ClpXP activity and protein synthesis in an *E. coli* extract-based cell-free expression system

**DOI:** 10.1038/s41598-018-21739-6

**Published:** 2018-02-22

**Authors:** Xinying Shi, Ti Wu, Christian M. Cole, Neal K. Devaraj, Simpson Joseph

**Affiliations:** Department of Chemistry and Biochemistry, University of California, San Diego, 9500 Gilman Drive, La Jolla, CA 92093-0314 USA

## Abstract

Protein degradation is a fundamental process in all living cells and is essential to remove both damaged proteins and intact proteins that are no longer needed by the cell. We are interested in creating synthetic genetic circuits that function in a cell-free expression system. This will require not only an efficient protein expression platform but also a robust protein degradation system in cell extract. Therefore, we purified and tested the activity of *E. coli* ClpXP protease in cell-free transcription-translation (TX-TL) systems that used *E. coli* S30 cell extract. Surprisingly, our studies showed that purified ClpXP added to the TX-TL system has very low proteolytic activity. The low activity of ClpXP was correlated with the rapid consumption of adenosine triphosphate (ATP) in cell extract. We improved the activity of ClpXP in cell extract by adding exogenous ATP and an energy regeneration system. We then established conditions for both protein synthesis, and protein degradation by ClpXP to occur simultaneously in the TX-TL systems. The optimized conditions for ClpXP activity will be useful for creating tunable synthetic genetic circuits and *in vitro* synthetic biology.

## Introduction

Protein degradation is an essential process in all living cells that is used to remove damaged or misfolded proteins and to tune gene expression by temporally controlling the concentration of regulatory proteins. In bacteria, targeted protein degradation is carried out by the AAA+ family of proteases, which include ClpAP, ClpXP, FtsH, HslUV (also known as ClpYQ), or Lon^1,2^. The mechanism of ClpXP is well understood and therefore ClpXP protein degradation is widely used in *in vitro* synthetic biology^3–5^. The ClpXP protease is an oligomer formed by 6 identical ClpX proteins, that form a hexameric ring, and 14 identical ClpP proteins, which form a double-ring structure^6,7^. The overall structure of the ClpXP protease resembles a barrel with a central axial pore. The ClpX protein uses ATP binding and hydrolysis to unfold protein substrates and translocate the denatured polypeptide through the central pore to the proteolytic chamber formed by the ClpP protease^1,2^. Most protein substrates that are targeted for degradation have a tag sequence that is recognized by a specific protease. In the case of ClpXP, protein substrates that have five distinct motifs (three located at the N-terminus and two at the C-terminus) are targeted for degradation^8^. Additionally, the *E. coli* ClpXP protease degrades proteins that have an 11-amino acid ssrA tag at the C terminus^9^. The ssrA tag is recognized by SspB, an adaptor protein that also binds to ClpX^10–12^. The interaction of SspB both with the ssrA tag and with ClpX enhances substrate recognition and improves degradation at lower substrate concentrations^10^.

We are interested in establishing dynamic genetic circuits using a cell-free transcription-translation (TX-TL) system. To create dynamic genetic circuits, it is essential to have robust protein degradation in the TX-TL system to provide a constant flux of proteins. Although previous studies have used ClpXP to target specific regulatory and reporter proteins for degradation *in vitro*, these studies used simple buffers to test the activity of ClpXP or used the endogenous ClpXP in the TX-TL system^3,4^. In these latter studies, the amount of active ClpXP in cell extract is low, resulting in a low rate of protein degradation^4^. To overcome this problem, one study added the genes coding for ClpX and ClpP to the TX-TL system to increase the concentration of the ClpXP protease in cell extract, which resulted in improved degradation of the target proteins^5^. Nevertheless, adding known concentration of highly active, purified ClpXP to the TX-TL system would offer greater flexibility in the design and modeling of dynamic gene circuits. Indeed a previous report showed that adding a purified, linked-hexameric version of ClpX to a TX-TL system increased the degradation of ssrA-tagged reporter proteins, indicating that this approach may be useful for creating dynamic gene circuits^13^.

Here we report the optimization of protein degradation in *E. coli* S30 cell extract-based TX-TL system by the ClpXP protease. To establish a protein degradation system that is highly active in the TX-TL system, we decided to use purified *E. coli* ClpXP protease. Our studies revealed that ClpXP activity is poor in the extract-based system because the concentration of ATP is low in the standard S30 cell extract used for TX-TL. Fortunately, adding ATP and an energy regeneration system significantly increased the activity of ClpXP in cell extract. We also demonstrate that ClpXP is active in cell extract enclosed inside phospholipid vesicles, suggesting that our work could have future application in the development of artificial cells. By using radioactive ATP, we analyzed how ATP was consumed and converted to other adenine nucleotides in either reaction buffers or in the TX-TL system under different reaction conditions. Finally, we optimized the TX-TL system so that both protein synthesis and protein degradation could occur simultaneously and efficiently. Our results suggest that TX-TL systems supplemented with known concentrations of ClpXP and an energy regeneration system could be used to create synthetic genetic circuits that function in vesicles, enabling robust and predictable protein degradation in artificial cells.

## Results

**Figure 1:**
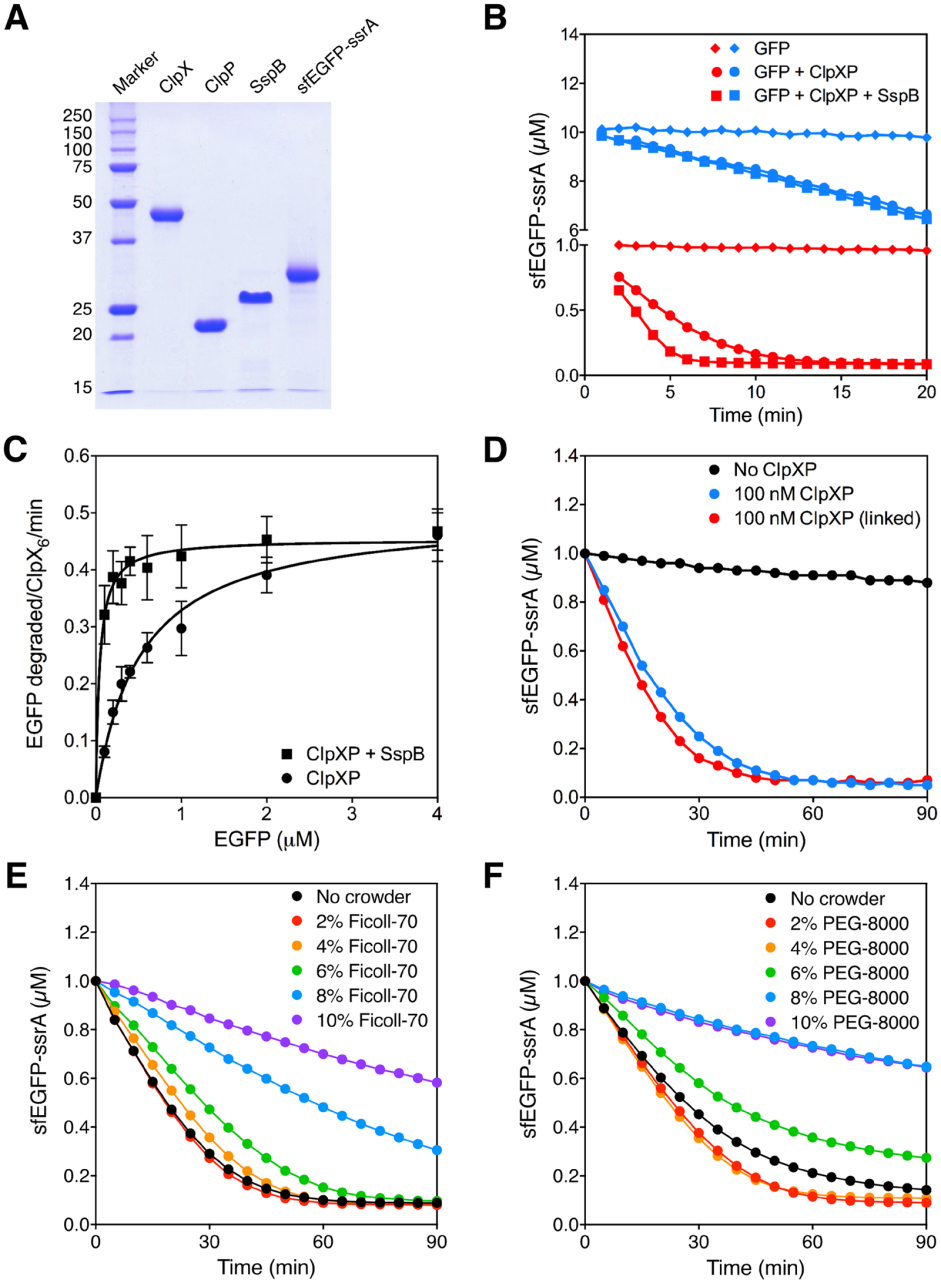
Degradation of sfEGFP-ssrA by ClpXP in buffer. (**A**) SDS-PAGE showing the purified *E. coli* ClpX, ClpP, SspB, and sfEGFP-ssrA proteins. (**B**) Degradation of sfEGFP-ssrA by ClpXP in the presence (squares) or in the absence (circles) of SspB performed in the PD buffer. The concentration of sfEGFP-ssrA was either 1 μM (red) or 10 μM (blue), and the concentration of ClpXP was 300 nM. No ClpXP was added to control reactions done in parallel (diamonds). (**C**) Measurement of steady-state kinetic parameters (*k*_*cat*_ and K_M_). The initial rate of protein degradation per ClpXP molecule at different sfEGFP-ssrA concentration was used to determine the kinetic parameters. The *k*_*cat*_ for ClpXP degradation of sfEGFP-ssrA is 0.46 ± 0.01 min^−1^ in the presence of SspB (square) and 0.50 ± 0.02 min^−1^ in the absence of SspB (circle). The K_M_ is 0.04 ± 0.007 μM in the presence of SspB and 0.52 ± 0.05 μM in the absence of SspB. (**D**) Protein degradation by the wild type ClpX (blue) and the linked-hexameric version (red) of ClpX_6_. The degradation of 1 μM sfEGFP-ssrA by 100 nM of each version of ClpXP. No ClpXP was added to the control reaction done in parallel (black). (**E**) Crowding effect of Ficoll-70 on ClpXP degradation. 1 μM sfEGFP-ssrA were degraded by 100 nM ClpXP in the presence of 0 to 10% w/w of crowding agent. (**F**) Crowding effect of PEG-8000 on ClpXP degradation. 1 μM sfEGFP-ssrA were degraded by 100 nM ClpXP in the presence of 0 to 10% w/w of crowding agent. The error bars show the standard deviation from at least three independent experiments. Plots without error bars are representative of at least three repeated experiments.

To establish the ClpXP protein degradation system, we overexpressed and purified C-terminal His-tagged ClpX, ClpP and SspB using *E. coli* **(**Fig. 1A**)**. We also constructed a vector to overexpress and purify the N-terminal His-tagged superfolder EGFP that has the ssrA tag sequence at the C-terminus (sfEGFP-ssrA). We used sfEGFP-ssrA as the substrate for the ClpXP protein degradation system. The sfEGFP-ssrA protein was highly expressed in *E. coli*, and the yield after purification was about 30 mg per liter culture. We analyzed the activity of the purified ClpXP system to degrade sfEGFP-ssrA by measuring the fluorescence intensity **(**Fig. 1B**)**. Additionally, we verified that the decrease in fluorescence intensity was due to protein degradation by ClpXP using SDS-PAGE (Supplementary Figure S1A). Steady-state kinetic parameters (*k*_cat_ and K_M_) were determined in the PD buffer. The *k*_cat_ for the degradation of sfEGFP-ssrA by ClpXP was 0.46 ± 0.01 min^−1^ in the presence of SspB and 0.50 ± 0.02 min^−1^ in the absence of SspB **(**Fig. 1C). The K_M_ was 0.043 ± 0.007 μM in the presence of SspB and 0.52 ± 0.06 μM in the absence of SspB. Thus, SspB is important for protein degradation by ClpXP when the concentration of the protein substrate (sfEGFP-ssrA) is low. These results are consistent with previously published data^10^.

Previous studies showed that a linked hexameric version of ClpX is more active in protein degradation *in vitro* than the monomeric wild type ClpX^13^. We purified the linked hexameric version of ClpX and compared its activity to the monomeric ClpX. The degradation of sfEGFP-ssrA by the wild type ClpXP and the linked ClpXP was monitored. The linked ClpXP was slightly more active (initial rates are 38 nM min^−1^ and 30 nM min^−1^, respectively) than the wild type ClpXP **(**Fig. 1D**)**. However, we decided to optimize the activity of the wild type ClpXP because the yield of the purified linked ClpXP was very low.

We also examined the macromolecular crowding effects on the proteolytic activity of ClpXP. Studies have shown that macromolecular crowders generally stimulate biochemical reactions^14–16^, and hence 2% PEG-8000 is a standard component in TX-TL system. Here we added two commonly used macromolecular crowders, Ficoll-70 and PEG-8000 up to 10% w/w to ClpXP reactions. Protein degradation by ClpXP was not affected by a low concentration of crowders but higher concentrations (larger than about 4%) decreased ClpXP activity **(**Fig. 1E,F**)**. It is possible that the crowded environment stabilizes sfEGFP-ssrA protein and slows down the unfolding process, which is the ATP-consuming step in ClpXP-dependent protein degradation. This may explain the inhibition of ClpXP activity at high concentrations of macromolecular crowders. Alternatively, high concentrations of crowders may cause protein precipitation or non-specific binding of proteins to the crowders resulting in the lowered ClpXP activity^16^. Nevertheless, we confirmed that 2% PEG-8000 present in TX-TL system won’t affect the performance of ClpXP.

**Figure 2:**
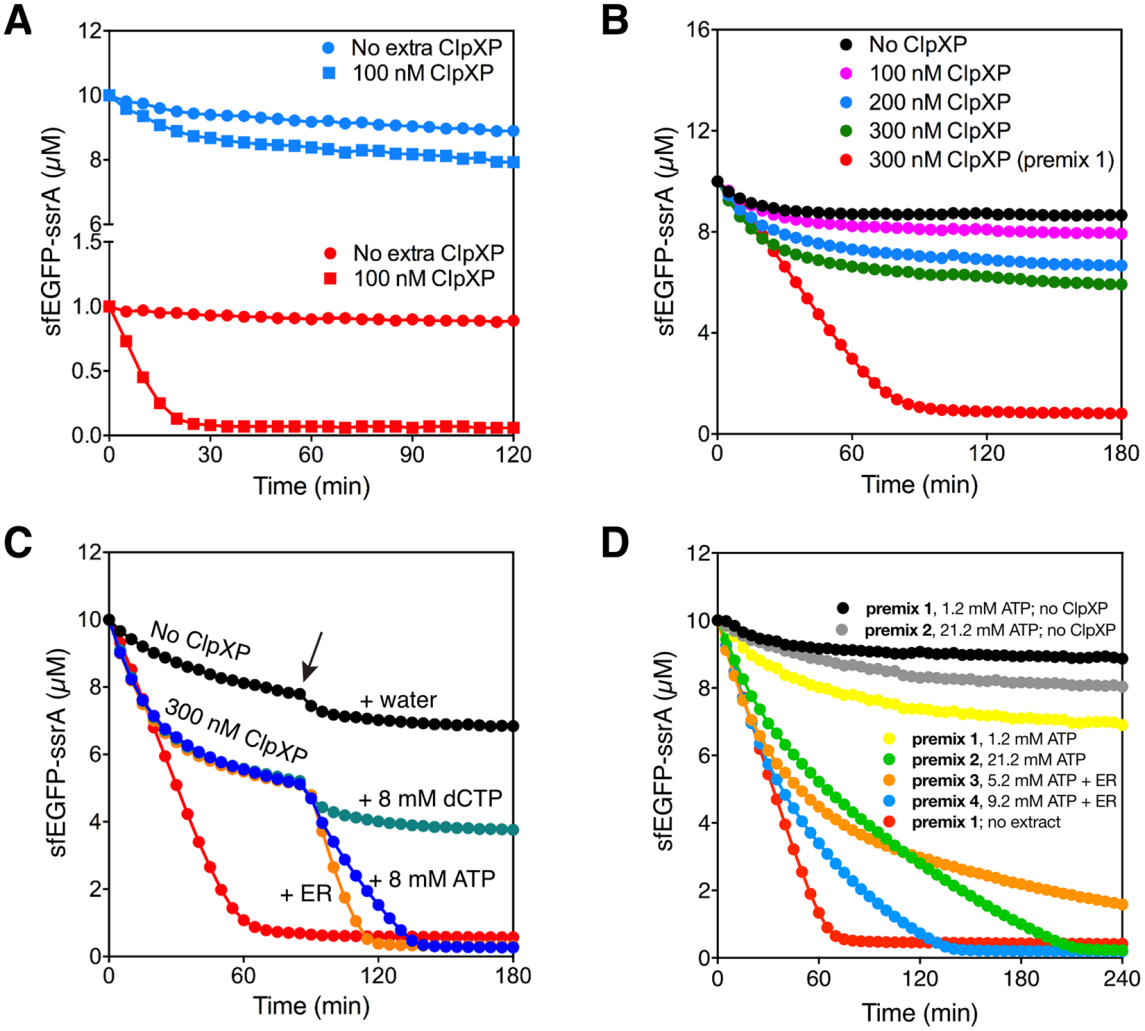
Optimization of ClpXP degradation in the TX-TL system. (**A**) ClpXP activity in the TX-TL system. The degradation of 1 μM (red) and 10 μM (blue) sfEGFP-ssrA by 100 nM extra ClpXP added to the complete TX-TL system (square) or by the endogenous ClpXP present in the TX-TL system (circle). In (**B**), (**C**) and (**D**) a positive control reaction was performed with 300 nM ClpXP and 10 µM sfEGFP-ssrA in premix 1 (red). (**B**) The degradation of sfEGFP-ssrA at different concentrations of ClpXP in the TX-TL system. 10 μM sfEGFP-ssrA were degraded by 0 nM (black), 100 nM (magenta), 200 nM (blue), or 300 nM (green) of ClpXP. (**C**) Analyzing the effect of increasing the ATP concentration on protein degradation by the wild type ClpXP in the TX-TL system. The degradation of 10 μM sfEGFP-ssrA by 0 nM (black), or 300 nM of ClpXP. After the degradation reactions were incubated for 1.5 hour, either 1 µL of water (magenta), 8 mM dCTP (green), 8 mM ATP (blue) or 8 mM ATP, 16 mM PEP, and 16 U/mL pyruvate kinase (indicated as ER) (orange) solution was added at the time point shown by the arrow. 1 µL of water was also added in negative control reaction (black) at the same time point. (**D**) Optimization of protein degradation by the wild type ClpXP in the TX-TL system. The degradation of 10 μM sfEGFP-ssrA by 300 nM ClpXP at different concentrations of ATP and in the presence of energy regeneration system. The standard TX-TL system with premix1 (yellow), premix 2 (orange), premix 3 (green), or premix 4 (blue). Control reactions were performed without ClpXP with premix 1 (black) and premix 2 (grey). Positive control reaction (red) was the degradation of 10 μM sfEGFP-ssrA with 300 nM ClpXP in premix 1 without cell extract. The final concentrations of ATP is 1.2 mM in premix 1, 21.2 mM in premix 2, 5.2 mM ATP in premix 3, and 9.2 mM in premix 4. Data are representative of at least three repeated experiments.

One of our long-term goals is to develop artificial cells that can execute dynamic genetic circuits. An essential feature for a dynamic genetic circuit is to have a sink to degrade the newly synthesized proteins without damaging the proteins required for TX-TL^5^. Targeted protein degradation could be achieved using the ClpXP protease. However, the *E. coli* S30 extract that we used for *in vitro* TX-TL has very low proteolytic activity from endogenous ClpXP and other proteases. **(**Fig. 2A**)**. Increasing the concentration of purified ClpXP protease in the *E. coli* S30 extract led to only a modest increase in the degradation of sfEGFP-ssrA **(**Fig. 2B**)**. We hypothesized that the energy in the system, 1.2 mM ATP and an energy regeneration system containing 30 mM phosphoenolpyruvate (PEP) and 48 U/mL pyruvate kinase (PK), was insufficient and limited the proteolytic activity of ClpXP in the TX-TL system. To verify this, we added either (1) 8 mM ATP or (2) 8 mM ATP plus extra energy regeneration components (16 mM PEP and 16 U/mL PK), 90 minutes after degradation reactions were started. As a control, we added water or dCTP (to account for Mg^2+^ chelation by ATP) to identical reactions performed in parallel. Addition of ATP or ATP plus energy regeneration components greatly stimulated the degradation of sfEGFP-ssrA and the reactions went nearly to completion **(**Fig. 2C**)**. These results showed that the concentration of ATP in the S30 cell extract must be increased for optimal ClpXP activity. *E. coli* cell extract contains some endogenous ClpX and ClpP^13^, so we next examined the amounts of additional ClpX and ClpP needed for optimal activity. Our results showed that degradation of 10 μM sfEGFP-ssrA was efficient with extra 300 nM ClpX and extra 100–300 nM ClpP in the presence of premix 4 (final concentration of ATP, PEP, and PK was 9.2 mM, 41 mM, and 58 U/ml respectively) in the TX-TL system. The proteolytic activity was mainly limited by ClpX concentration but additional ClpP was required to speed up protein degradation when 300 nM ClpX was used^17^, suggesting that there’s more endogenous ClpP than ClpX (Supplementary Figure S1C,D). To further improve the activity of ClpXP in the cell-free expression systems, we added the energy regeneration system and titrated the concentration of ATP in the degradation reaction. Our studies showed that the S30 extract with 9.2 mM ATP, 41 mM PEP and 58 U/mL PK resulted in efficient degradation of sfEGFP-ssrA by ClpXP **(**Fig. 2D**)**. We also verified that the decrease in fluorescence intensity was due to protein degradation by ClpXP using Western blot (Supplementary Figure S1B). It’s also worth noting that although degradation was efficient and almost complete, it was still slower than in buffer control. Finally, all premixes were tested without adding purified ClpXP to serve as negative controls and see if they were activating the endogenous ClpXP. The results with different premixes but no ClpXP added were similar, which indicated that additional energy components won’t activate endogenous ClpXP significantly.

**Figure 3:**
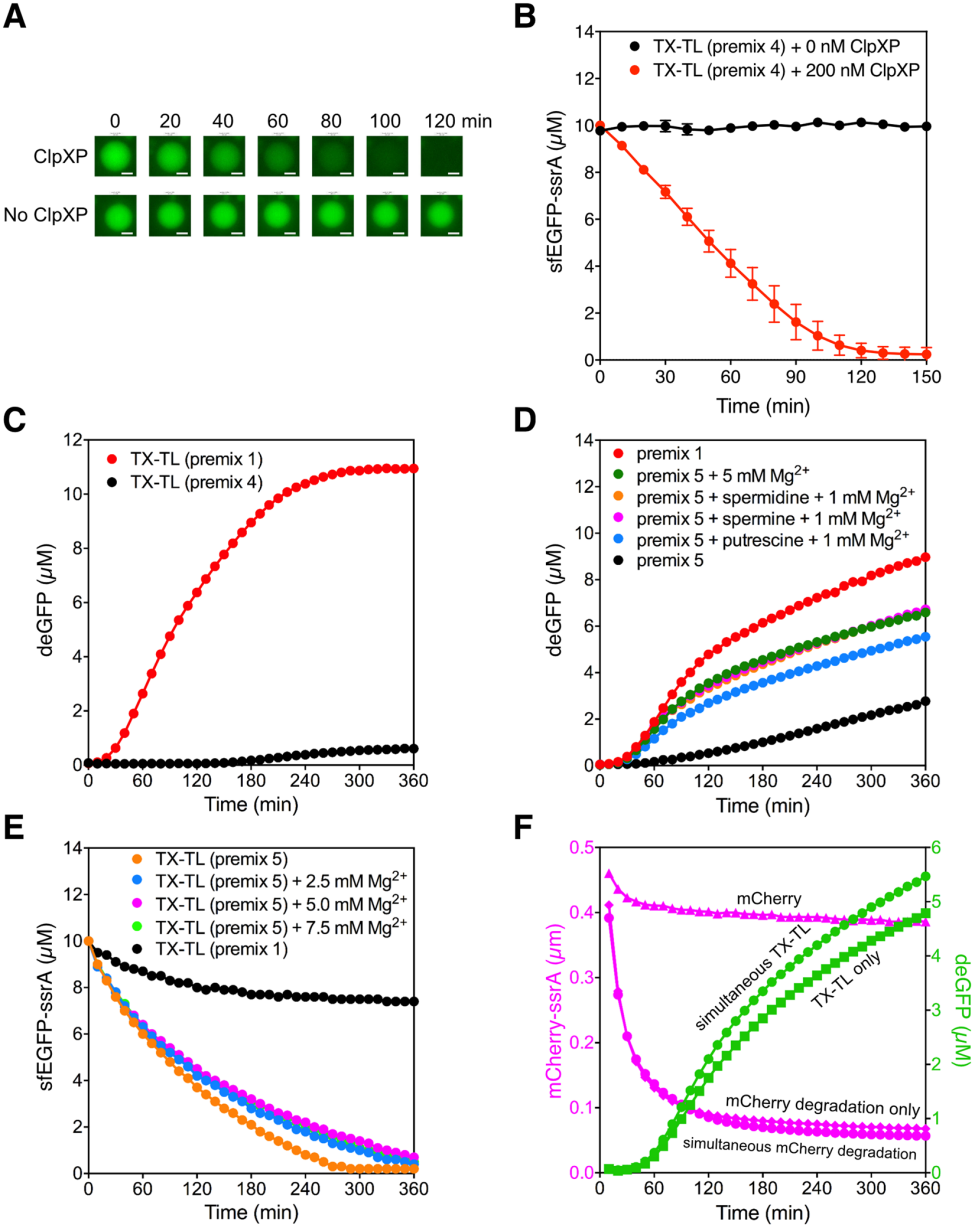
Degradation of sfEGFP-ssrA in vesicles by ClpXP and optimizing both TX-TL and ClpXP activity. (**A**) Fluorescence images of sfEGFP-ssrA degradation in a representive vesicle at 0, 20, 40, 60, 80, 100, 120 minutes. The degradation of 10 μM sfEGFP-ssrA in the TX-TL cell extract with 200 nM ClpXP added (top), or no ClpXP added (bottom) are shown. The scale bars represent 10 µm. (**B**) Time course of ClpXP degradation in vesicles. The degradation kinetics of sfEGFP-ssrA in the presence (red) or absence of ClpXP (black) in vesicles. The degradation kinetics of at least 10 vesicles were measured to calculate the standard deviations. (**C**) Protein synthesis in the optimized condition for ClpXP activity. Reactions were performed in TX-TL systems containing premix 1 (red) or premix 4 (black). (**D**) Effects of Mg^2+^ and polyamines on TX-TL reactions. Reactions were performed under standard condition with premix 5 and additional Mg-glutamate and/or polyamines: 5 mM Mg-glutamate (green); 2 mM spermidine and 1 mM Mg-glutamate (orange); 0.5 mM spermine and 1 mM Mg-glutamate (magenta); 8 mM putrescine and 1 mM Mg-glutamate (blue). Control reactions were performed with premix 1 (red) and premix 5 (black). (**E**) Effect of additional Mg^2+^ on ClpXP activity in TX-TL systems. Control reaction (black) was performed in premix 1. Premix 5 was used for all the other reactions. The extra Mg-glutamate added to the reactions were: 0 mM (orange), 2.5 mM (blue), 5 mM (red), and 7.5 mM (green). (**F**) Simultaneous protein synthesis and degradation in the TX-TL system. All the reactions were performed under standard condition with premix 5 and extra 5 mM Mg-glutamate. Control reactions for protein degradation were performed with 500 nM mCherry-ssrA without ClpXP (pink triangles) or with 300 nM ClpXP (pink diamonds); TX-TL reaction control for deGFP synthesis was performed in the absence of mCherry-ssrA and ClpXP (green squares); combined reactions included degradation of 500 nM mCherry-ssrA by 300 nM ClpXP (pink circles) and deGFP synthesis (green circles). Data are representative of at least three repeated experiments.

Since one of our long-terms goals is to reconstitute genetic circuits in artificial cells, we tested the degradation of sfEGFP-ssrA by ClpXP in lipid bound vesicles. The lipid used to form vesicles was 1-palmitoyl-2-oleoyl-sn-glycero-3-phosphocholine (POPC) and vesicles were formed by the inverse-emulsion method^18–20^. The vesicles contained S30 extract with 10 μM sfEGFP-ssrA and 200 nM ClpXP. Degradation of sfEGFP-ssrA was monitored by fluorescence imaging using a spinning disk confocal microscope. ClpXP completely degraded sfEGFP-ssrA in about 2 hours, demonstrating that ClpXP was still very active in the vesicles (Fig. 3A,B). This is an important first step for building dynamic genetic circuits in vesicles.

Another essential feature to enable a dynamic genetic circuit is for the synthesis of target proteins simultaneous to protein degradation. This requires that conditions must be optimized for both protein degradation and protein synthesis. Therefore, we tested the efficiency of TX-TL under the optimized ClpXP degradation condition. The reporter protein synthesized is deGFP, an N-terminal truncated, C-terminal modified version of enhanced green fluorescent protein (eGFP), which has been shown to have better expression yield in TX-TL^21^. Unfortunately, TX-TL of deGFP was almost fully inhibited in the optimized condition for ClpXP degradation (Fig. 3C). We tested whether degradation by ClpXP could occur at conditions that were more similar to the TX-TL reaction by titrating the concentration of ATP, and presence or absence of PEP and PK (Supplementary Figure S2A,B). These experiments showed that efficient degradation of 10 µM sfEGFP-ssrA by ClpXP occurs when an additional 4–8 mM ATP is added **(**Supplementary Figure S2A**)**. Additionally, it is not essential to add extra PEP or PK to the ClpXP degradation reaction because they are present in sufficient concentrations in the premix buffer **(**Supplementary Figure S2B**)**. Thus, protein degradation by ClpXP in the S30 extract was efficient if the concentration of ATP is 7.2–9.2 mM (premix 1 has 1.2 mM ATP and we added extra 6–8 mM ATP).

In contrast, the TX-TL reaction normally has 1.2 mM ATP, and 7.2–9.2 mM ATP inhibits the reaction. We reasoned that the extra ATP that we added to improve the activity of ClpXP was chelating Mg^2+^ thereby lowering the concentration of free Mg^2+^ causing the inhibition of TX-TL. Indeed, a recent study showed that nearly half of the Mg^2+^ in the cell is chelated by nucleotides^22^. We tested whether TX-TL activity could be restored by adding extra Mg^2+^. Increasing the concentration of Mg^2+^ resulted only in a modest 30% increase in TX-TL compared to the control reaction without extra Mg^2+^ **(**Supplementary Figure S3A**)**. We titrated the concentration of ATP, Mg^2+^, PEP, and PK to further optimize the TX-TL reaction **(**Supplementary Figure S3B–F**)**. We also showed that the polyamines, such as spermine, putrescine, and spermidine, could partly substitute for the Mg^2+^ in the TX-TL reaction **(**Supplementary Figure S4**)**. The addition of an extra 5 mM Mg-glutamate (final Mg^2+^ concentration is 19.2 mM), or 0.5 mM spermine and 1 mM Mg-glutamate, or 2 mM spermidine and 1 mM Mg-glutamate increased the activity of the TX-TL reaction to ~70% of the maximum. (Fig. 3D). ClpXP was stored in buffer supplemented with 10% glycerol. Therefore, the optimized TX-TL reaction has 2.5% final glycerol concentration, which could be responsible for the ~30% inhibition in gene expression **(**Supplementary Figures S5A,B). The optimized conditions (with extra 6 mM ATP and 5 mM Mg-glutamate) were then used to test the activity of ClpXP again. ClpXP activity was slightly slowed by the addition of extra 5 mM Mg-Glutamate (Fig. 3E). Thus, we established new optimized conditions (7.2 mM ATP and 19.2 mM Mg^2+^ final concentrations) in which both the TX-TL reaction and protein degradation by ClpXP can occur simultaneously, although it is not the most efficient condition for each individual reaction. The optimized conditions were used to test whether the synthesis of deGFP and the degradation of mCherry-ssrA by ClpXP could occur simultaneously. As shown in Fig. 3F, we observed the efficient synthesis of deGFP and the degradation of mCherry-ssrA in the same reaction confirming that the optimized conditions are suitable for the design and testing of gene circuits *in vitro*.

**Figure 4:**
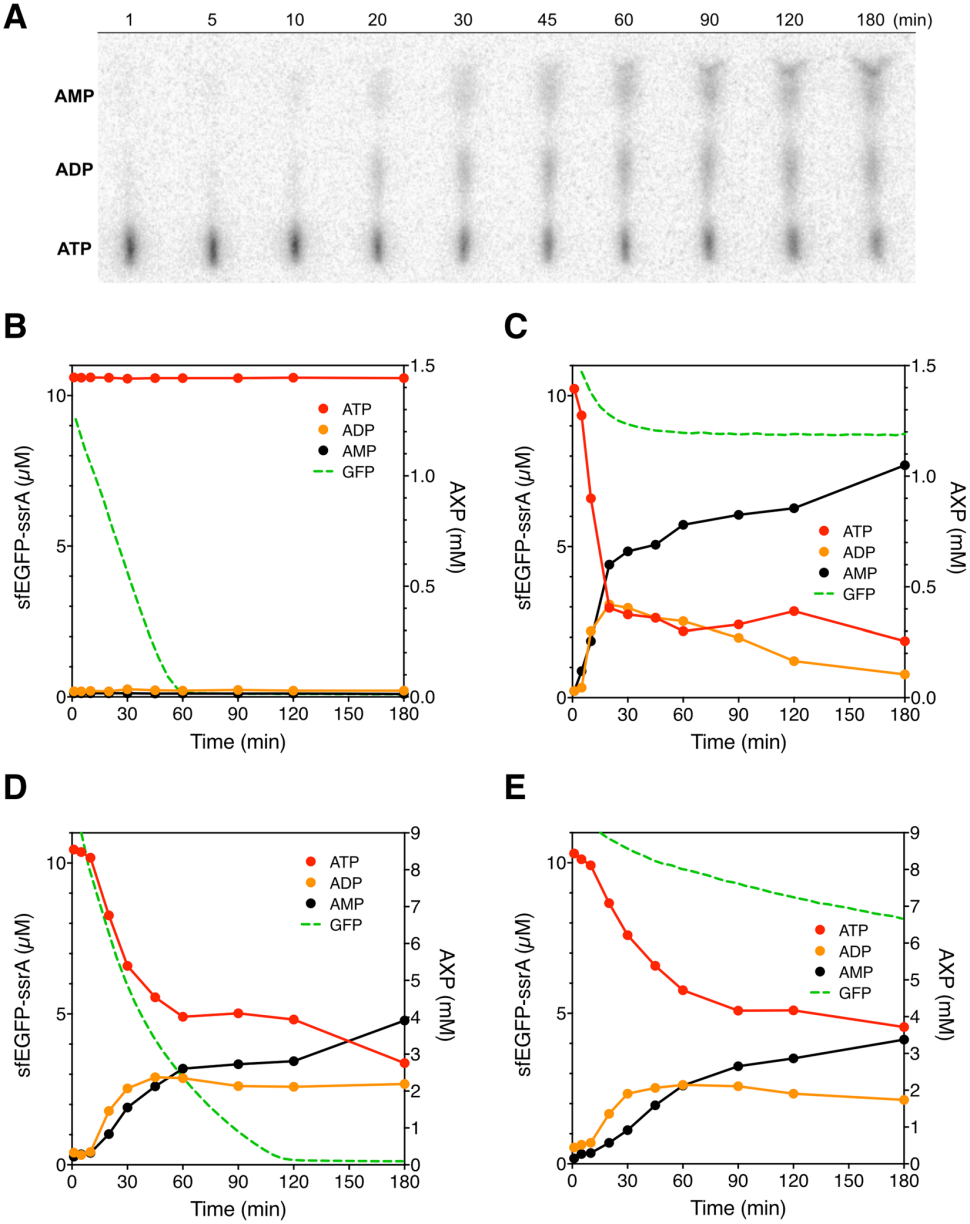
ATP consumption and protein degradation by ClpXP. (**A**) A representative phosphor image of ATP consumption assay by ClpXP in the TX-TL system with additional 8 mM ATP, 16 mM phosphoenolpyruvate, and 16 U/mL pyruvate kinase. Samples were collected and quenched at 1, 5, 10, 20, 30, 45, 60, 90, 120, and 180 minutes after the reaction started. ATP, ADP, and AMP were separated on a PEI-cellulose TLC plate and quantified with a phosphor imager. The dark spots correspond to radioactive ATP, ADP, and AMP, respectively, from bottom to top. Spots were quantified and normalized by each lane and the ratio of each adenosine phosphate species were plotted. (**B**–**E**) are graphs showing protein degradation and ATP consumption. All reactions were performed with 300 nM ClpXP (except in (**E**)) and 10 µM sfEGFP-ssrA in different conditions for 3 hours. (**B**) The reaction was performed in premix 1. (**C**) The reaction was performed in the TX-TL system with premix 1. (**D**) The reaction was performed in the TX-TL system with premix 4. (**E**) The reaction was performed without ClpXP in the TX-TL system with premix 4. Data are representative of at least three repeated experiments.

To better understand why the same energy regeneration system and ATP concentration, which was enough to saturate 300 nM ClpXP (Supplementary Figure S1E), gave us very different ClpXP activity in buffers and the TX-TL systems, we established an ATP consumption assay using PEI-cellulose TLC to monitor the concentrations of [α-^32^P]-ATP and its hydrolyzed products (Fig. 4A). The degradation of sfEGFP-ssrA by ClpXP was performed in the presence of [α-^32^P]-ATP and the degradation reaction was continuously monitored by the decrease in the fluorescence intensity of sfEGFP-ssrA. In parallel, an aliquot of the degradation reaction was used to monitor ATP consumption at fixed time points. As a control reaction, we used ClpXP with premix 1, which shows zero-order-like reaction kinetics for protein degradation, and a stable level of ATP concentration (Fig. 4B), demonstrating that the energy regeneration system can maintain the ATP concentration. When we used premix 1 without any PK, the enzyme responsible for regenerating ATP by transferring a phosphate group from PEP to ADP, protein degradation slowed down quickly, and ATP concentration decreased as ADP concentration increased. This result shows that the stable level of ATP in the reaction with premix 1 is maintained by the ATP regeneration mechanism of PK (Supplementary Figure S5C**)**. Thus, ClpXP is very active in premix 1 which has 1.2 mM ATP and an efficient energy regeneration system with 25 mM PEP and 42 U/mL PK.

However, one of the problems we encountered originally is that the activity of ClpXP decreases drastically in the TX-TL system. We discovered that ATP was consumed rapidly in the TX-TL system, and simultaneously high levels of AMP accumulated (Fig. 4C and Supplementary Figure S5D). This behavior was not observed in the buffer systems (Fig. 4B and Supplementary Figure S5C). Even with the creatine kinase-phosphocreatine energy regeneration system, ATP was consumed quickly, while AMP accumulated to high levels **(**Supplementary Figure S5E). In the improved system with premix 4 (extra 8 mM ATP, 16 mM PEP, 16 U/mL PK were added, and final concentrations are 9.2 mM ATP, 41 mM PEP, and 58 U/mL PK), we can see that ATP concentration went down in the first hour and then reached a steady-state level concentration of about 4 mM (Fig. 4D) while ADP concentration also reached a steady-state level, and sfEGFP-ssrA is degraded at a reasonably rapid rate. ATP concentration went down again after two hours as AMP concentration went up. Interestingly, byproducts from ATP hydrolysis, such as AMP, inorganic phosphate, and pyrophosphate, do not inhibit ClpXP (Supplementary Figure S6A–C), but the concentration of ADP or the [ATP]/[ADP] ratio are more important for optimal activity in the buffer system **(**Supplementary Figure S6E**)**. Finally, we found that more than 50% of the ATP was consumed in the first hour in cell extract even without the addition of exogenous ClpXP (Fig. 4E). Our results demonstrate that ATP is mostly consumed by enzymes present in the *E. coli* S30 cell extract. Therefore, for optimal degradation of proteins by ClpXP in the TX-TL system, the steady-state concentration of ATP must be maintained high enough by adding more ATP or a more efficient energy regeneration system.

## Discussion

Protein degradation plays a key role for controlling gene expression by removing regulatory proteins that are no longer needed by the cell. Studies have shown that protein degradation is also important for spatially and temporally coupling synthetic genetic circuits in cells^23,24^. However, the activity of the endogenous proteases in cell extract is low and insufficient to build dynamic *in vitro* genetic circuits.

In a previous study, purified ClpXP was used to degrade ssrA-tagged eGFP in bulk and in vesicles, but the reaction was performed in a simple buffer^3^. In another study, the degradation rate by the endogenous ClpXP present in the *E. coli* S30 extract was analyzed with 1 µM of deGFP-ssrA substrate^4^. The rate of degradation by the endogenous ClpXP was slow (*v*_*o*_ = 10 nM min^−1^) and inadequate to create dynamic genetic circuits. To overcome this shortcoming, a subsequent study cloned the *clpP-clpX* genes from *E. coli* into a vector under the P70a promoter^5^. The vector was added at different concentrations to the TX-TL cell extract and preincubated to produce ClpX and ClpP proteins. Then eGFP-ssrA protein was added as a substrate and the reaction was monitored. A high rate of protein degradation was observed with 6 nM plasmid and 5 µM eGFP-ssrA. Thus, the low activity of the endogenous ClpXP was overcome by synthesizing ClpXP in the TX-TL system, and this approach could be used to create simple gene circuits *in vitro*^5^.

However, synthesizing ClpXP from vectors by the TX-TL system has some disadvantages. First, determining the precise concentration of ClpXP in the reaction will require additional experiments. Second, the production of ClpXP in parallel by the TX-TL system may interfere with the function of complex genetic circuits. Therefore, we decided to purify the *E. coli* ClpXP protease and test its activity in a TX-TL cell extract. Consistent with previous studies, the activity of the purified ClpXP was high in the PD buffer but was very low when added to the standard S30 cell extract used for TX-TL^3,4^. Our results show that the low activity of the purified ClpXP is due to the low steady-state concentration of ATP in the *E. coli* S30 cell extract used for TX-TL, which has 1.2 mM ATP and an energy regeneration system (25 mM PEP and 42 U/mL PK) for supporting efficient protein synthesis. Importantly, increasing the final concentration of ATP to 9.2 mM, PEP to 41 mM, and PK to 58 U/mL in the TX-TL significantly improved the activity of the purified ClpXP. The degradation of sfEGFP-ssrA in the S30 cell extract went to completion under the optimized reaction conditions. This indicates that protein degradation by ClpXP consumes lots of ATP and the steady-state level of ATP in the cell extract is insufficient to support robust protein degradation. Indeed, previous studies have shown that ClpXP hydrolyzes about 600 ATPs to degrade one molecule of the I27 domain of human titin (121 residues)^25^. We also show that ClpXP is active in cell extract inside vesicles. The rate of sfEGFP-ssrA degradation by ClpXP in vesicles is similar to the rate observed in bulk indicating that vesicles containing cell extract optimized for gene expression and protein degradation could be used to create artificial cells that execute dynamic genetic circuits.

We analyzed how energy consumption and regeneration affect the activity of ClpXP. First, we showed that our energy regeneration system using PEP/PK is efficient that we can even use ADP as the initial energy molecule (Supplementary Figure S6D). We also confirmed that AMP and other byproducts do not directly inhibit ClpXP. So the correlation between the accumulation of AMP and the lower activity of ClpXP could be accounted by the decreasing total amount of energy molecules, including both ATP (for reaction) and ADP (for regenerating ATP). The accumulation of AMP is probably due to some enzymes in the cell extract, e.g. adenylate kinase, ADP-dependent NAD(P)HX dehydratase, and aminoacyl tRNA synthetases. Inhibiting these enzymes or finding a way to regenerate ATP or ADP from AMP could potentially help ClpXP to work better in the extract-based cell-free systems^26,27^.

Moreover, we noticed that the steady-state level of ATP concentration in the optimized conditions (Fig. 4D) was about 4 mM in the TX-TL system; however, the rates of protein degradation by ClpXP were slower compared to the reaction with 1.2 mM ATP in the premix buffer **(**Fig. 4B**)**. This suggests that ATP concentration is not the only factor to affect the activity of ClpXP in our cell-free systems. Additional experiments showed that the rates of protein degradation by ClpXP decreased when we added higher concentration of ADP to achieve lower [ATP]/[ADP] ratio in the buffer without energy regeneration system (Supplementary Figure S6E). This is because protein degradation by ClpXP is mainly powered by the ATPase domains on the ClpX subunit and changes in the [ATP]/[ADP] ratio is expected to affect the activity of ClpXP significantly.

Our studies showed that protein degradation by ClpXP in the S30 extract needs a higher concentration of ATP than is optimal for the TX-TL reaction. We systematically optimized the concentration of ATP, Mg^2+^, PK, and PEP to identify a new condition that is optimal for both ClpXP activity and the TX-TL reaction. The new optimized condition is 7.2 mM ATP, 19.2 mM Mg^2+^, 25 mM PEP, and 42 U/mL PK. We used the optimized condition to successfully carry out simultaneous synthesis of deGFP and the degradation of mCherry-ssrA by ClpXP. Although the new conditions are not optimal for the individual reactions, we have identified conditions where the activity of ClpXP and the TX-TL reaction can be altered easily by changing the steady-state concentration of ATP or Mg^2+^. Our results implicate that to establish dynamic genetic circuits containing multiple energy consuming biochemical reactions, such as protein synthesis or protein degradation by AAA+ proteases, the supply and turnover of energy molecules should be accounted with care. We showed that it’s possible to achieve high efficiency in both protein synthesis and protein degradation in extract-based cell-free systems. More complicated dynamic genetic circuits could be optimized based on the same principle, and we believe that the ability to modulate the rates of protein synthesis and protein degradation will be very useful for fine tuning the performance of *in vitro* genetic circuits.

## Methods

### Cloning and protein purification

*E. coli clpX*, *clpP*, and *sspB* genes were subcloned in pMCSG26 vector with C-terminal His-tag. sfEGFP-ssrA and mCherry-ssrA were subcloned in pMCSG7 vector with N-terminal His-tag. Plasmid pACYC-FLAG-dN6-His encoding ClpX linked hexamer version was from Addgene (# 22143). *E. coli* BL21 (DE3) cells were used to overexpress and purify all proteins. Cells were grown at 37 °C to A_600_ 0.5–0.8, chilled to 18 °C for ClpX, chilled to 30 °C for ClpP and SspB, chilled to 37 °C for sfEGFP-ssrA and mCherry-ssrA. Protein overexpression was induced with 1 mM IPTG and the cells were grown for another 3–4 hours. The cells were lysed using a French press at 18000 psi and the proteins purified by Ni-NTA affinity chromatography as previously described^28,29^. Purified ClpP, and SspB proteins were dialyzed against storage buffer (50 mM Tris-HCl, pH 7.5, 200 mM KCl, 25 mM MgCl_2_, 1 mM DTT, 10% Glycerol). ClpX was purified further using a Superdex 75 16/60 column equilibrated in the above storage buffer. The concentrations of ClpX_6_, ClpP_14_, and SspB_2_ were 1.4 μM (hexamer), 12 μM(tetradecamer), 57 μM(dimer), respectively. sfEGFP-ssrA was dialyzed against buffer (20 mM K-Hepes, pH 7.5). mCherry-ssrA were stored in buffer containing 50 mM Tris-HCl, pH 7.5, 150 mM NaCl. The concentrations of sfEGFP-ssrA and mCherry-ssrA were 120 μM and 125 μM, respectively. ClpX linked hexamer protein was purified by Ni-NTA affinity chromatography and further purified using a Superdex 200 16/60 size exclusion column (GE Healthcare) and stored in the buffer containing 50 mM Tris-HCl, pH7.6, 300 mM NaCl, 1 mM DTT, 10% glycerol. The concentration of the linked ClpX_6_ was 3 μM. Protein concentrations were measured using Bradford method. All proteins were stored at −80 °C.

### Fluorescent protein measurement and quantification

The fluorescence of sfEGFP-ssrA or deGFP was measured by using Tecan GENios microplate reader (excitation filter wavelength (band width): excitation filter 485 (20) nm; emission filter wavelength (band width): 535 (25) nm; gain 45) unless stated otherwise. Fluorescence intensity was transformed into concentration unit using a standard curve made with our purified sfEGFP-ssrA. Data was analyzed and graphs were created by Graphpad Prism software. All TX-TL and degradation reactions were performed at 30 °C.

### *In vitro* protein degradation assay

Degradation assay of sfEGFP-ssrA by ClpXP was performed with 10 µL reaction volume in PD buffer (25 mM K-Hepes, pH 7.6, 200 mM KCl, 5 mM MgCl_2_, 0.032% Nonidet P-40, and 10% glycerol) with an energy regeneration system (4 mM ATP, 16 U/mL creatine kinase, and 16 mM creatine phosphate). If SspB was used, the concentration of SspB was the same as sfEGFP-ssrA and SspB was pre-incubated with sfEGFP-ssrA for the degradation assay. 100 nM ClpXP (ClpX_6_:ClpP_14_ = 1:3) and 1 µM sfEGFP-ssrA was used for most degradation assay in PD buffer because we could analyze the function of SspB in the reaction and easily follow the time course of the reaction. Adenosine nucleotides, phosphates, and pyrophosphates were prepared as 100 mM stock solution at pH 7.0 and added separately to desired concentration. Adenosine 5′-monophosphate sodium salt (AMP, prod# A1752), adenosine 5′-diphosphate monopotassium salt dehydrate (ADP, prod# A5285), and sodium pyrophosphate decahydrate (PPi, prod# 221368) were purchased from Sigma Aldrich, and 85% phosphoric acid was purchased from Macron Chemicals (prod# 2796–05). The effect of ADP and AMP on ClpXP activity were analyzed in premix 1. Fluorescence of sfEGFP-ssrA was monitored by microplate reader. 10 µM sfEGFP-ssrA degradation by 300 nM ClpXP was analyzed using SDS-PAGE.

### *k*_*cat*_ and K_M_ measurement

*k*_*cat*_ and K_M_ measurement was performed in PD buffer by combining 50 nM ClpXP with varied concentration of sfEGFP-ssrA. Degradation rates were calculated from the initial linear loss of fluorescence. Curve fitting was performed using the Michaelis-Menten equation built in Graphpad Prism software. The experiment was repeated two times.

### Macromolecular crowding effect on ClpXP activity

Degradation were performed in PD buffer system with 1 µM sfEGFP-ssrA, 100 nM ClpXP, and various crowders, including up to 20% w/w PEG-400, PEG-8000, PEG-20000, Ficoll-70, and Ficoll-400. Reactions were performed in 10 µL of volume on a 384-well plate and fluorescence of sfEGFP-ssrA was monitored by using microplate reader.

### Optimization of ClpXP activity in cell extract

*E. coli* Rosetta 2 (DE3) cell extract was prepared as described previously^30^ with minor modifications. Briefly, 1 g of cells were resuspended in 1.1 mL of S30A buffer. The cells were lysed by passing it twice through a French Press at 18000 psi. The extract was clarified by centrifugation at 12000 g for 10 min. The clear supernatant was preincubated at 37 °C for 80 min and the extract was clarified again at 12000 g for 10 min. The clear supernatant was dialyzed against 1 L of S30B buffer containing 60 mM K-glutamate and 14 mM Mg-glutamate for 3 hours. The extract was clarified at 12000 g for 10 min and was stored as small aliquots at −80 °C. Degradation reactions were performed in 12 µL final volume consisting of 4 µL cell extract (final protein concentration is 7.5–8 mg/mL), 3 µL ClpXP (final concentration 300 nM) or buffer contains 25 mM MgCl_2_ and 200 mM KCl, 1 µL sfEGFP-ssrA (at different concentrations), 4 µL premix 1 [42 mM K-Hepes, pH 8.2, 0.7 mM CTP and UTP, 1.2 mM GTP and ATP, 25 mM phosphoenol pyruvate (PEP), 42 U/mL pyruvate kinase (PK), 2.5 mM of 20 amino acid, 1.7% PEG-8000, 0.057 mM folinic acid, 0.17 mg/mL tRNA, 0.8 mM putrescine, 8 mM Magnesium glutamate, 120 mM potassium glutamate, all at final concentrations]^30^. The ClpX_6_ to ClpP_14_ ratio was 1:1 in the reactions with cell extract and 1:1.3 in the reactions without cell extract. Additionally, the ClpXP degradation in cell extract was optimized by increasing the concentration of ATP, PEP and PK as follows: premix 2 (extra 20 mM ATP was added, and final ATP concentration is 21.2 mM), premix 3 (extra 4 mM ATP, 16 mM PEP, 16 U/mL PK were added, and final concentrations are 5.2 mM ATP, 41 mM PEP and 58 U/mL PK), and premix 4 (extra 8 mM ATP, 16 mM PEP, 16 U/mL PK were added, and final concentrations are 9.2 mM ATP, 41 mM PEP and 58 U/mL PK). Premix 5 (extra 6 mM ATP was added to premix 1. The final ATP concentration is 7.2 mM). The differences in the concentrations of ATP, PEP, PK, and Mg^2+^ in the premixes are shown in Table S1. 10 µM sfEGFP-ssrA degradation by 300 nM ClpXP was analyzed using Western blot with anti-His tag antibody purchased from Thermo Scientific (Cat# MA1-21315).

### *In vitro* Transcription-Translation (TX-TL) of deGFP

Standard TX-TL reactions were composed of 4 µL S30 extract (final protein concentration is 7.5–8 mg/mL), 1 µL pBEST-deGFP plasmid (Addgene plasmid# 40019, final concentration is 8.3 nM), 4 µL premix 1 (final concentration of ATP is 1.2 mM), and 3 µL of ClpXP storage buffer (which contributes 50 mM KCl, 6.2 mM MgCl_2_ and 2.5% glycerol to the final reaction). Reactions were performed in 12 µL of volume on a 384-well plate and fluorescence of deGFP was monitored by microplate reader.

### Optimization of TX-TL system under ClpXP degradation condition

Optimization of TX-TL system under the ClpXP degradation condition were performed in the presence of premix 5 (final concentration of ATP is 7.2 mM). Extra Mg-Glutamate or polyamines were added to restore the activity. Reactions were performed in 12 µL volume containing of 4 µL cell extract (final concentration of protein is 7.5–8 mg/mL), 4 µL premix 5 (final concentration of ATP is 7.2 mM), and 1 µL pBEST-deGFP plasmid (final concentration is 8.3 nM), 3 µL of ClpXP storage buffer (which contributes 50 mM KCl and 6.2 mM MgCl_2_ to the final reaction), and extra Mg^2+^ and/or polyamines.

### ClpXP proteolytic activity under optimized TX-TL condition

ClpXP reactions were performed in 12 µL final volume consisting of 4 µL cell extract (final protein concentration is 7.5–8 mg/mL), 4 µL premix 5 (final concentration of ATP is 7.2 mM), 3 µL ClpXP (final concentration 300 nM) and magnesium glutamate (at different concentration), 1 µL sfEGFP-ssrA (final concentration is 10 µM).

### Simultaneous protein synthesis and degradation in TX-TL systems

Protein synthesis and ClpXP reactions were performed in 12 µL final volume consisting of 4 µL cell extract (final protein concentration is 7.5–8 mg/mL), 4 µL premix 5 with pBEST-deGFP plasmid (final concentrations are: 7.2 mM ATP, 19.2 mM Mg^2+^ and 5 nM plasmid), 3 µL ClpXP (final concentration 300 nM), and 1 µL mCherry-ssrA (final concentration is 0.5 µM). Reactions were performed at 30 °C on a 384-well plate and the fluorescence of deGFP (excitation filter wavelength (band width): 485 (20) nm, emission filter wavelength (band width): 535 (25) nm) and mCherry-ssrA (excitation filter wavelength (band width): 560 (10) nm and emission filter wavelength (band width): 612 (20) nm) were simultaneously monitored by using Tecan Infinite F200 microplate reader.

### ATP consumption assay

This method was modified from Rajagopal and Lorsch’s protocol of ATP and GTP Hydrolysis Assay (TLC)^31^. PEI-cellulose TLC plate was cut to the appropriate size, pre-developed completely with water, and then dried. After setting up reactions with [α-^32^P]-ATP, 3 µL reaction solution was taken out and mixed with 3 µL quenching solution (1 M HCOOH) at different time points. In addition, 9 µL reaction solution of each sample was taken out right after mixing and transferred to a 384-well plate for parallel measurement of protein degradation. When samples from all time points were collected, 1 µL of each quenched sample was spotted on the marked places on the plate, and air dried. The TLC plate was developed with 0.5 M K_2_HPO_4_, pH 3.5, for 25 min, then dried. The TLC plate was wrapped in a clear plastic wrap, and exposed to a storage phosphor screen (Amersham Bioscience). The screen was then scanned with Bio-Rad Personal Molecular Imager™ (PMI™) System or GE Healthcare Life Sciences Typhoon FLA 9500, and the data was analyzed with the Bio-Rad Quantity One^®^ or ImageJ software.

### Vesicle preparation

Vesicles were prepared with POPC (1-palmitoyl-2-oleoyl-sn-glycero-3-phophocholine) purchased from Avanti Inc. (cat# 850457 P) as described previously^18–20^. To prepare the lipid suspension for the inner leaflet, POPC was first dissolved in chloroform, and the chloroform was subsequently evaporated with dry nitrogen in the hood affording a lipid film. The POPC film was then re-dissolved in heavy mineral oil (Fisher Scientific cat# O122-1) at 5 mg/mL, heated at 50 °C, and sonicated for one hour in a water bath to evenly distribute the lipid. 10 μL of degradation reaction (described in next section) was added to 100 µL of POPC oil and vigorously mixed until it was a cloudy emulsion formed. The emulsion was then layered over 100 µL of 0.5 M Tris-HCl, pH 7.5, centrifuged for 10 min at 9000 rcf at room temperature. The bilayer vesicles were formed as they passed through the water/oil phase and pelleted at the bottom. Oil and some buffer were discarded, and the vesicles were resuspended in the rest of buffer.

### Degradation assay in vesicles

Degradation reaction was prepared by mixing 200 nM ClpXP and 10 μM sfEGFP-ssrA in optimized TX-TL system (plus extra 8 mM ATP, 16 mM phosphoenolpyruvate, and 16 U/mL pyruvate kinase). Vesicles were then prepared via the inverse emulsion method using the degradation reaction mixture as previously described. 10 µL of the resulting vesicle solution was added to a glass microscope slide and covered with a glass coverslip supported with vacuum grease. Fluorescence microscope images were acquired on a Yokagawa spinning disk system (Yokagawa, Japan) built around an Axio Observer Z1 motorized inverted microscope (Carl Zeiss Microscopy GmbH, Germany) with a 63×, 1.40 NA oil immersion objective to an ORCA-Flash 4.0 camera managed using Micromanager software. 10–20 vesicles were selected and pictures were taken every 10 min using a 488 nm, 100 mW OPSL laser, to excite the sfEGFP. Fluorescence intensity was quantified by using ImageJ software.

### Data availability

The datasets generated and/or analyzed during the current study are available from the corresponding author on reasonable request.

## Electronic supplementary material

Supplementary Information

## References

[1] Sauer RT, Baker TA (2011). AAA+ proteases: ATP-fueled machines of protein destruction. Annu Rev Biochem.

[2] Baker TA, Sauer RT (2012). ClpXP, an ATP-powered unfolding and protein-degradation machine. Biochim Biophys Acta.

[3] Noireaux V, Bar-Ziv R, Godefroy J, Salman H, Libchaber A (2005). Toward an artificial cell based on gene expression in vesicles. Phys Biol.

[4] Shin J, Noireaux V (2010). Study of messenger RNA inactivation and protein degradation in an Escherichia coli cell-free expression system. J Biol Eng.

[5] Garamella J, Marshall R, Rustad M, Noireaux V (2016). The All E. coli TX-TL Toolbox 2.0: A Platform for Cell-Free Synthetic Biology. ACS Synth Biol.

[6] Glynn SE, Martin A, Nager AR, Baker TA, Sauer RT (2009). Structures of asymmetric ClpX hexamers reveal nucleotide-dependent motions in a AAA+ protein-unfolding machine. Cell.

[7] Wang J, Hartling JA, Flanagan JM (1997). The structure of ClpP at 2.3 A resolution suggests a model for ATP-dependent proteolysis. Cell.

[8] Flynn JM, Neher SB, Kim YI, Sauer RT, Baker TA (2003). Proteomic discovery of cellular substrates of the ClpXP protease reveals five classes of ClpX-recognition signals. Mol Cell.

[9] Gottesman S, Roche E, Zhou Y, Sauer RT (1998). The ClpXP and ClpAP proteases degrade proteins with carboxy-terminal peptide tails added by the SsrA-tagging system. Genes Dev.

[10] Levchenko I, Seidel M, Sauer RT, Baker TA (2000). A specificity-enhancing factor for the ClpXP degradation machine. Science.

[11] Flynn JM, Levchenko I, Sauer RT, Baker TA (2004). Modulating substrate choice: the SspB adaptor delivers a regulator of the extracytoplasmic-stress response to the AAA+ protease ClpXP for degradation. Genes Dev.

[12] Bolon DN, Grant RA, Baker TA, Sauer RT (2004). Nucleotide-dependent substrate handoff from the SspB adaptor to the AAA+ ClpXP protease. Mol Cell.

[13] Sun, Z., Kim, J., Singhal, V. & Murray, R. M. Protein degradation in a TX-TL cell-free expression system using ClpXP protease. *bioRxiv*, (2015).

[14] Rivas G, Minton AP (2016). Macromolecular Crowding *In Vitro*, *In Vivo*, and In Between. Trends Biochem Sci.

[15] Sharp KA (2016). Unpacking the origins of in-cell crowding. Proc Natl Acad Sci USA.

[16] Ge X, Luo D, Xu J (2011). Cell-free protein expression under macromolecular crowding conditions. PLoS One.

[17] Jendrasiak GL, Hasty JH (1974). The electrical conductivity of hydrated phospholipids. Biochim Biophys Acta.

[18] Pautot S, Frisken BJ, Weitz DA (2003). Engineering asymmetric vesicles. Proc Natl Acad Sci USA.

[19] Pautot S, Frisken BJ, Weitz DA (2003). Production of unilamellar vesicles using an inverted emulsion. Langmuir.

[20] Chowdhuri S, Cole CM, Devaraj NK (2016). Encapsulation of Living Cells within Giant Phospholipid Liposomes Formed by the Inverse-Emulsion Technique. Chembiochem.

[21] Shin J, Noireaux V (2010). Efficient cell-free expression with the endogenous E. Coli RNA polymerase and sigma factor 70. J Biol Eng.

[22] Pontes MH, Sevostyanova A, Groisman EA (2015). When Too Much ATP Is Bad for Protein Synthesis. J Mol Biol.

[23] Prindle A (2014). Rapid and tunable post-translational coupling of genetic circuits. Nature.

[24] Elowitz MB, Leibler S (2000). A synthetic oscillatory network of transcriptional regulators. Nature.

[25] Kenniston JA, Baker TA, Fernandez JM, Sauer RT (2003). Linkage between ATP consumption and mechanical unfolding during the protein processing reactions of an AAA+ degradation machine. Cell.

[26] Itoh H, Kawazoe Y, Shiba T (2006). Enhancement of protein synthesis by an inorganic polyphosphate in an E. coli cell-free system. J Microbiol Methods.

[27] Resnick SM, Zehnder AJ (2000). *In vitro* ATP regeneration from polyphosphate and AMP by polyphosphate:AMP phosphotransferase and adenylate kinase from Acinetobacter johnsonii 210A. Appl Environ Microbiol.

[28] Davis JH, Baker TA, Sauer RT (2009). Engineering synthetic adaptors and substrates for controlled ClpXP degradation. J Biol Chem.

[29] Kim YI, Burton RE, Burton BM, Sauer RT, Baker TA (2000). Dynamics of substrate denaturation and translocation by the ClpXP degradation machine. Mol Cell.

[30] Thompson J, Dahlberg AE (2004). Testing the conservation of the translational machinery over evolution in diverse environments: assaying Thermus thermophilus ribosomes and initiation factors in a coupled transcription-translation system from Escherichia coli. Nucleic Acids Res.

[31] Rajagopal V, Lorsch JR (2013). ATP and GTP hydrolysis assays (TLC). Methods Enzymol.

