# Supplementary material for: Optimization of ClpXP activity and protein synthesis in an E. coli extract-based cell-free expression system
Source: Sci Rep. 2018 Feb 22;8:3488. doi: 10.1038/s41598-018-21739-6 (PMC5823941; doi:10.1038/s41598-018-21739-6)
Supplement: Supplementary file 1 — Supplementary Information [file 41598_2018_21739_MOESM1_ESM.pdf]

# Optimization of ClpXP activity and protein synthesis in an *E. coli* extract based cell-free expression system

Xinying Shi, Ti Wu, Christian Cole, Neal Devaraj, and Simpson Joseph

Department of Chemistry and Biochemistry,
University of California, San Diego,
9500 Gilman Drive, La Jolla, CA 92093-0314.

*Correspondence to:*
*Simpson Joseph*
*4102 Urey Hall,*
*Department of Chemistry and Biochemistry,*
*University of California, San Diego,*
*9500 Gilman Drive,*
*La Jolla, CA 92093-0314.*

*Phone: (858) 822-2957*
*Fax: (858) 534-7042*
*E-mail: sjoseph@ucsd.edu*

## Supporting Information

**Supplementary Figure S1. Degradation of sfEGFP-ssrA.** (A) SDS-PAGE showing the degradation of 10 μM sfEGFP-ssrA by 100 nM ClpXP in the PD buffer system. Samples were collected at 0-, 1-, 2-, and 3-hour time points. (B) Western Blot showing degradation of 10 μM sfEGFP-ssrA degradation by 300 nM ClpXP in the cell extracts with premix 4. Samples were collected at 0-, 1-, 2-, and 3-hour time points. (C) and (D) The degradation of 10 μM sfEGFP-ssrA at different concentrations of ClpX and ClpP in the TX-TL system with premix 4. (C) sfEGFP-ssrA were degraded in the presence of no extra ClpX with addition of no extra ClpP (red, square), 100 nM nM ClpP (blue, square), 200 nM ClpP (green, square) and 300 nM ClpP (orange, square) or 100 nM ClpX with addition of no extra ClpP (red, circle), 100 nM ClpP (blue, circle), 200 nM ClpP (green, circle) and 300 nM ClpP (orange, circle). A control reaction (black) with no extra ClpX and ClpP was performed in the TX-TL system. (D) sfEGFP-ssrA were degraded in the presence of 200 nM ClpX with addition of no extra ClpP (red, square), 100 nM ClpP (blue, square), 200 nM ClpP (green, square) and 300 nM ClpP (orange, square), or 300 nM ClpX with addition of no extra ClpP (red, circle) 100 nM ClpP (blue, circle), 200 nM ClpP (green, circle) and 300 nM ClpP (orange, circle). (E) Titration of ATP concentrations in protein degradation assay. 10 μM sfEGFP-ssrA was degraded by 300 nM ClpXP in premix buffers supplying 0 mM (red), 0.3 mM (orange), 1 mM (yellow), 1.5 mM (green), 3 mM (cyan), and 9 mM (blue) of ATP. Data are representative of three repeated experiments.

**Supplementary Figure S2. Optimization of the ClpXP activity under TX-TL condition.** The concentration of ClpXP was 300 nM and the concentration of sfEGFP-ssrA was 10 μM in all reactions. (A) and (B) Degradation of sfEGFP-ssrA by ClpXP in the presence of different

concentrations of ATP, PEP and PK. Control reactions in premix 1with and without lysate are shown in red and black colors, respectively. The reactions are labeled 1 to 11 and the final concentrations of ATP, PEP, PK, and $Mg^{2+}$ are shown in the Table S1. Data are representative of three repeated experiments.

**Supplementary Figure S3. Optimization of protein synthesis under the ClpXP degradation reaction condition by adding magnesium glutamate.** All reactions were carried out in premix 1 with different combinations of extra ATP, PEP, PK, and Mg-glutamate. Control reaction (red) were performed in the standard condition with premix 1 (final concentrations are 1.2 mM ATP, 25 mM PEP, 41 mM PK, and 14.2 mM total $Mg^{2+}$). Optimization of protein synthesis were based on the extra ATP, PEP and PK added to premix 1. (A) TX-TL reactions with extra 8 mM ATP, 16 mM PEP, and 16 U/mL PK (final concentrations are 9.2 mM ATP, 41 mM PEP, and 58 U/mL PK). The concentrations of the extra Mg-glutamate added are: 0 mM (black), 7.5 mM (blue), 10 mM (green), and 12.5 mM (magenta). (B) TX-TL reactions with extra 4 mM ATP, 16 mM PEP, and 16 U/mL PK (final concentrations are 5.2 mM ATP, 41 mM PEP, and 58 U/mL PK). The concentrations of the extra Mg-glutamate added are: 0 mM (black), 7.5 mM (blue), 10 mM (green), and 12.5 mM (magenta). (C) TX-TL reactions with extra 8 mM ATP and 16 U/mL PK (final concentrations are 9.2 mM ATP, and 58 U/mL PK). The concentrations of the extra Mg-glutamate added to the reactions are: 0 mM (black), 7.5 mM (blue), 10 mM (green), and 12.5 mM (magenta). (D) TX-TL reactions with extra 8 mM ATP and 16 mM PEP (final concentrations are 9.2 mM ATP, and 41 mM PEP). The concentrations of the extra Mg-glutamate added to the TX-TL reactions are: 0 mM (black), 7.5 mM (blue), 10 mM (green), and 12.5 mM (magenta). (E) TX-TL reactions with extra 8 mM ATP (final concentration is 9.2 mM ATP). The concentrations of the

extra Mg-glutamate added are: 0 mM (black), 7.5 mM (blue), 10 mM (green), and 12.5 mM (magenta). (F) TX-TL reactions with extra 6 mM ATP (final concentration is 7.2 mM ATP). The concentrations of the extra Mg-glutamate added are: 0 mM (black), 7.5 mM (blue), 10 mM (green), and 12.5 mM (magenta). Data are representative of three repeated experiments.

**Supplementary Figure S4. Optimization of protein synthesis under ClpXP degradation condition by adding polyamines.** All reactions were carried out in the standard condition with premix 5 (final concentrations are 7.2 mM ATP, and 14.2 mM total $Mg^{2+}$) with different concentrations of extra polyamines or combinations of extra polyamine and Mg-glutamate. Control TX-TL reactions were performed in premix 5 with extra 5 mM (black) or 0 mM (grey) Mg-glutamate added. (A) Optimization with spermine. The concentration of spermine in the TX-TL reactions are: 0.0625 mM (magenta), 0.125 mM (purple), 0.25 mM (green), 0.5 mM (blue), and 1 mM (orange). (B) Optimization with 0.5 mM spermine and extra Mg-glutamate. The concentrations of the extra Mg-glutamate added to the TX-TL reactions are: 1 mM (magenta), 2 mM (purple), 3 mM (green), 4 mM (blue), and 5 mM (orange). (C) Optimization with putrescine. The concentration of putrescine in the TX-TL reaction are: 4 mM (magenta), 6 mM (purple), 8 mM (green), 10 mM (blue), and 12 mM (orange). (D) Optimization with 8 mM putrescine and extra Mg-Glutamate. The concentrations of the extra Mg-glutamate added to the TX-TL reactions are: 1 mM (magenta), 2 mM (purple), 3 mM (green), 4 mM (blue), and 5 mM (orange). (E) Optimization with spermidine. The concentration of spermidine in the TX-TL reactions are: 0.25 mM (magenta), 0.5 mM (purple), 1 mM (green), 2 mM (blue), and 4 mM (orange). (F) Optimization with 2 mM spermidine and extra Mg-Glutamate. The concentrations of the extra

Mg-glutamate added to the TX-TL reactions are: 1 mM (magenta), 2 mM (purple), 3 mM (green), 4 mM (blue), and 5 mM (orange). Data are representative of three repeated experiments.

**Supplementary Figure S5. Effects of glycerol on TX-TL protein synthesis, and ATP consumption assays.** (A) Effect of glycerol on gene expression in the TX-TL system. The expression of deGFP in the TX-TL system with premix 1 in the presence of 0% (red); 2.5% (blue); 5% (orange); and 7.5% (green) glycerol. (B) Glycerol inhibits deGFP expression in the TX-TL system with premix 5. TX-TL reactions without glycerol (red), and with 2.5% glycerol (black). (C) (D) (E) are graphs showing protein degradation and ATP consumption assays. All reactions were carried out with 300 nM ClpXP and 10 μM sfEGFP-ssrA in different conditions for 3 hours. (C) The reaction was carried out in premix 1 without any pyruvate kinase. (D) The reaction was carried out without ClpXP in the TX-TL system without additional pyruvate kinase in the premix 1. (E) The reaction was carried out in the TX-TL system with premix 1, and CP/CK replaced PEP/PK for energy regeneration. (F) Comparison of protein degradation with PEP/PK and CP/CK energy regeneration systems in both buffer and in the TX-TL system. Data are representative of three repeated experiments.

**Supplementary Figure S6. Effects of AMP and ADP on protein degradation by ClpXP.** (A) Reaction with AMP. (B) Reaction with AMP and two equivalent of phosphoric acid (Pi). (C) Reaction with AMP and one equivalent of disodium pyrophosphorate (PPi). All reactions were carried out in premix 1 which has 1.5 mM ATP. Control reactions were performed with 0 nM (black) or 300 nM ClpXP (red). (D) Effects of ADP on the activity of ClpXP. Total

concentration of [ATP] + [ADP+Pi] was fixed at 1.5 mM, but the ratio of [ATP] to [ADP+Pi] was varied as follows: 1.5 mM ATP only (red), [ATP]:[ADP+Pi] = 2:1 (orange), [ATP]:[ADP+Pi] = 1:1 (yellow), [ATP]:[ADP+Pi] = 1:2 (green), 1.5 mM ADP+Pi only (cyan). (E) Effects of [ATP]/[ADP] ratio on the activity of ClpXP. Reactions with 4 mM ATP and 0 to 4 mM ADP+Pi were performed in the absence of any energy regeneration system. Data are representative of three repeated experiments.

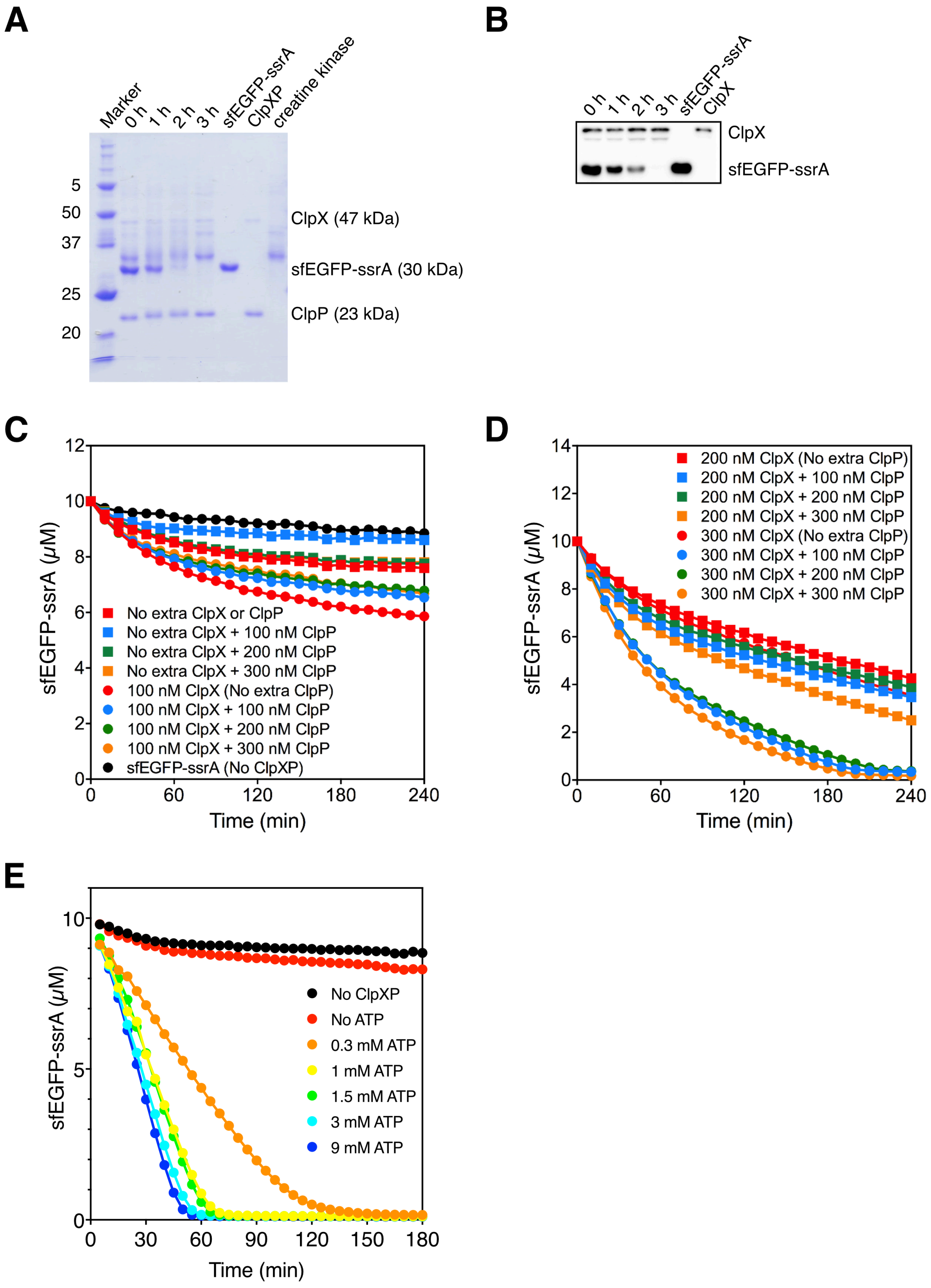


Supplementary Figure S1

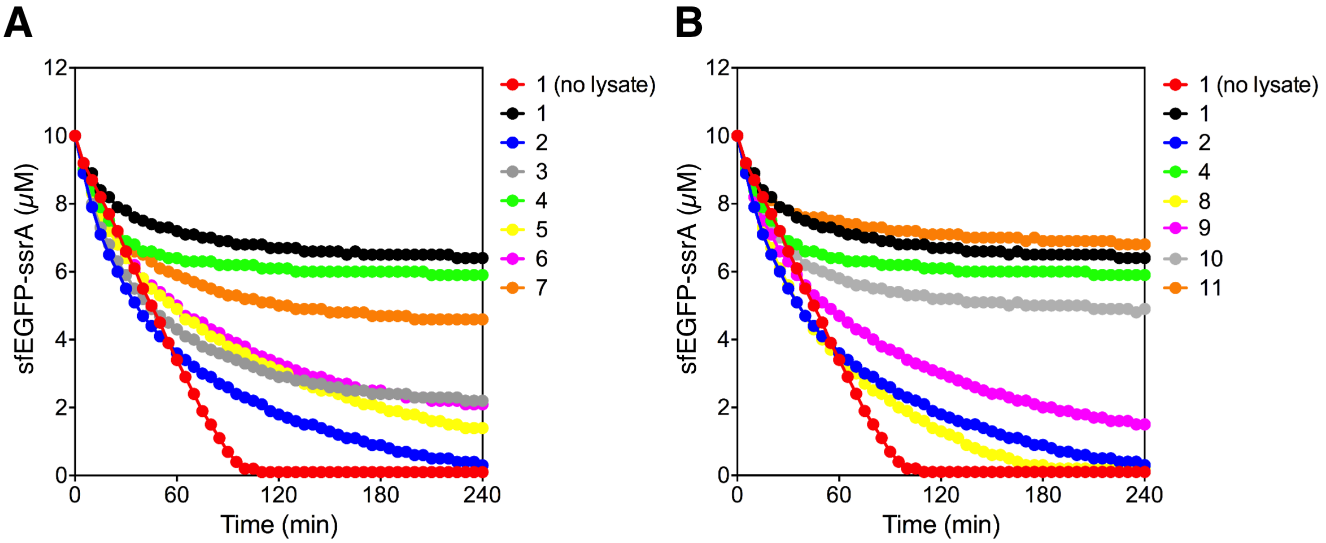


**Table S1 Final concentration of the energy regeneration system and $Mg^{2+}$**

| Reactions | ATP (mM) | PEP (mM) | PK (U/mL) | $Mg^{2+}$ (mM) |
|---|---|---|---|---|
| 1 | 1.2 | 25 | 42 | 14.2 |
| 2 | **9.2** | **41** | **58** | 14.2 |
| 3 | **5.2** | **41** | **58** | 14.2 |
| 4 | 1.2 | **41** | **58** | 14.2 |
| 5 | **9.2** | 25 | 42 | 14.2 |
| 6 | **7.2** | 25 | 42 | 14.2 |
| 7 | **5.2** | 25 | 42 | 14.2 |
| 8 | **9.2** | **41** | 42 | 14.2 |
| 9 | **9.2** | 25 | **58** | 14.2 |
| 10 | 1.2 | **41** | 42 | 14.2 |
| 11 | 1.2 | 25 | **58** | 14.2 |

Changes from reaction 1 are indicated in bold

Supplementary Figure S2

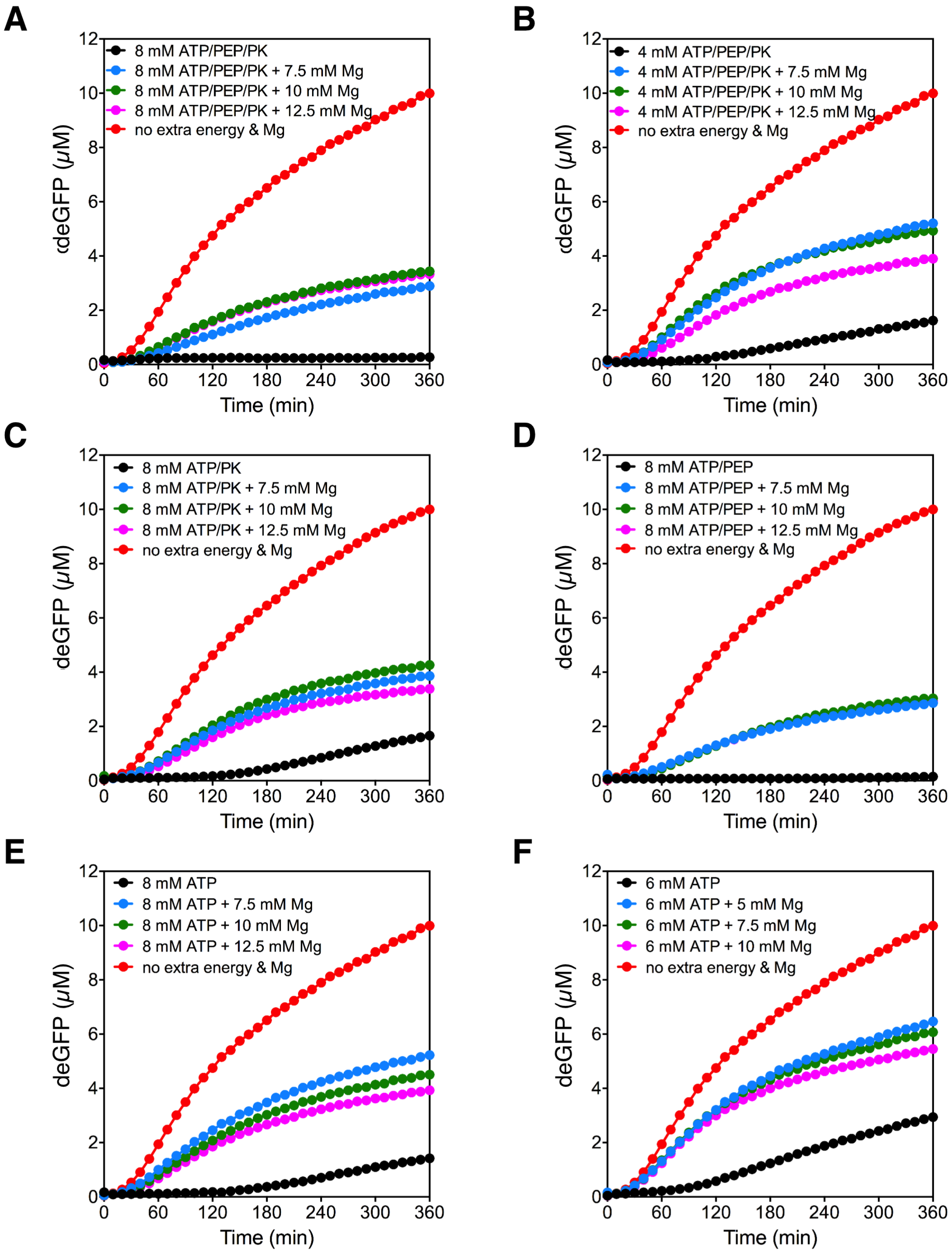


Supplementary Figure S3

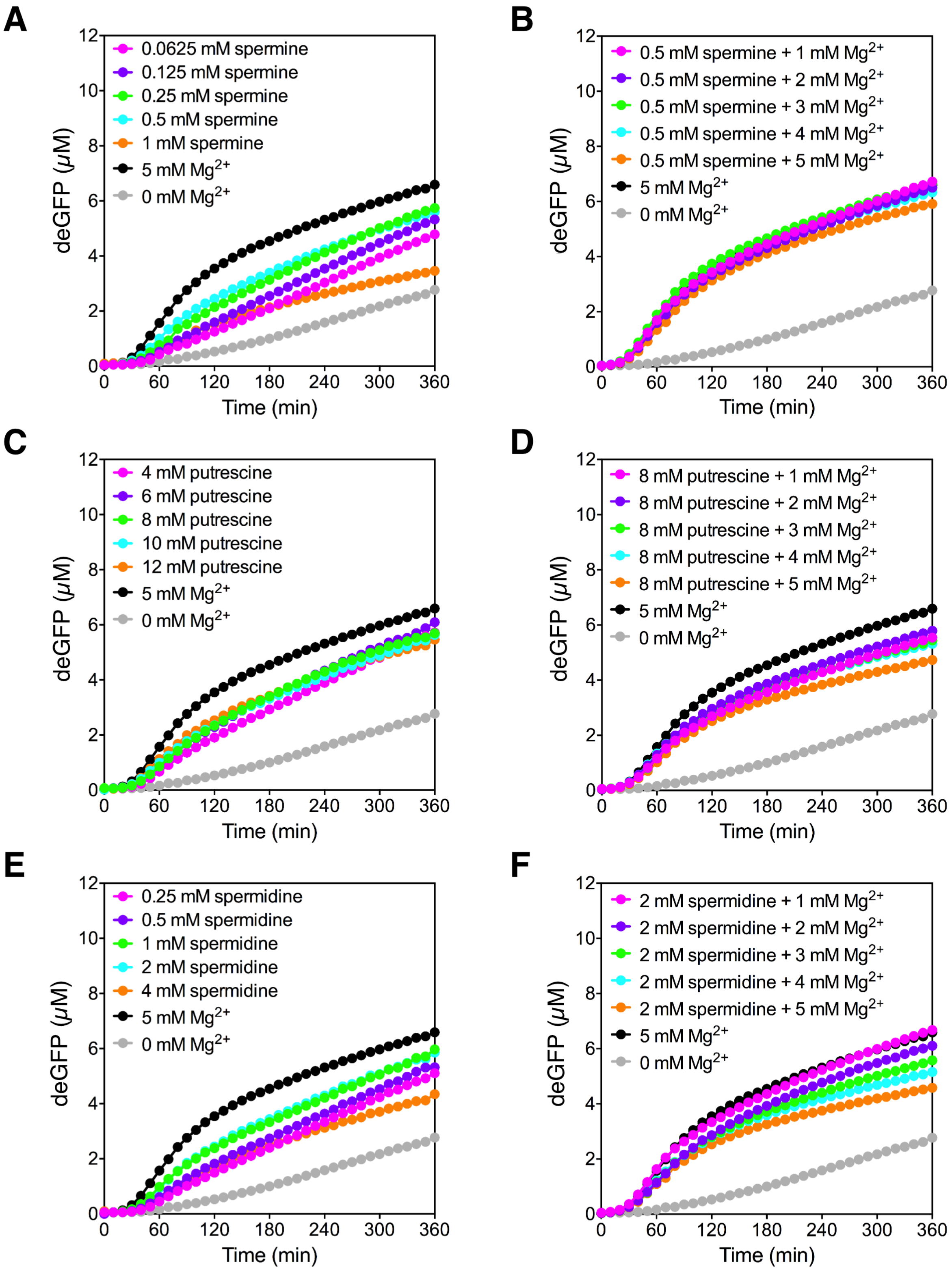


Supplementary Figure S4

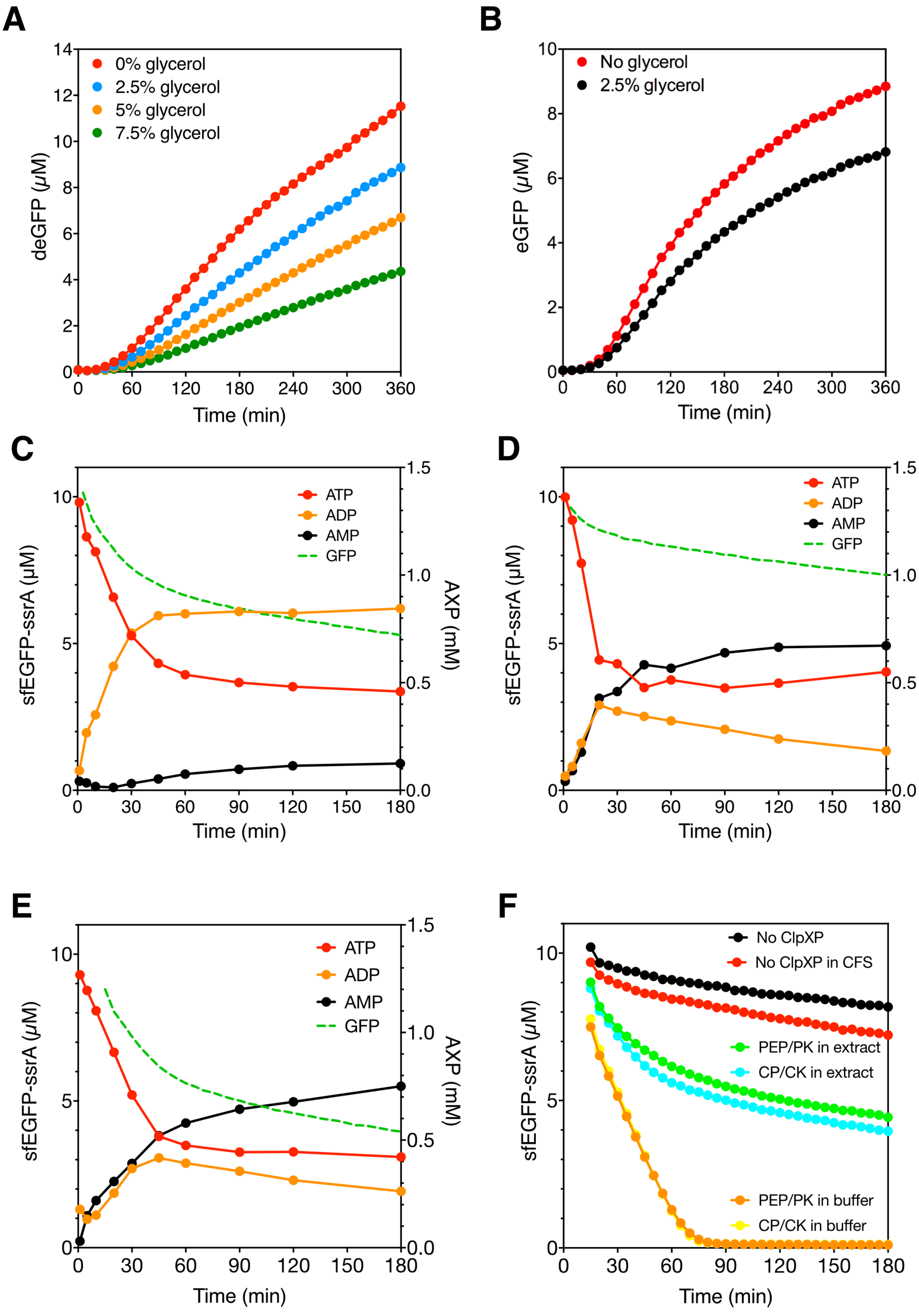


Supplementary Figure S5

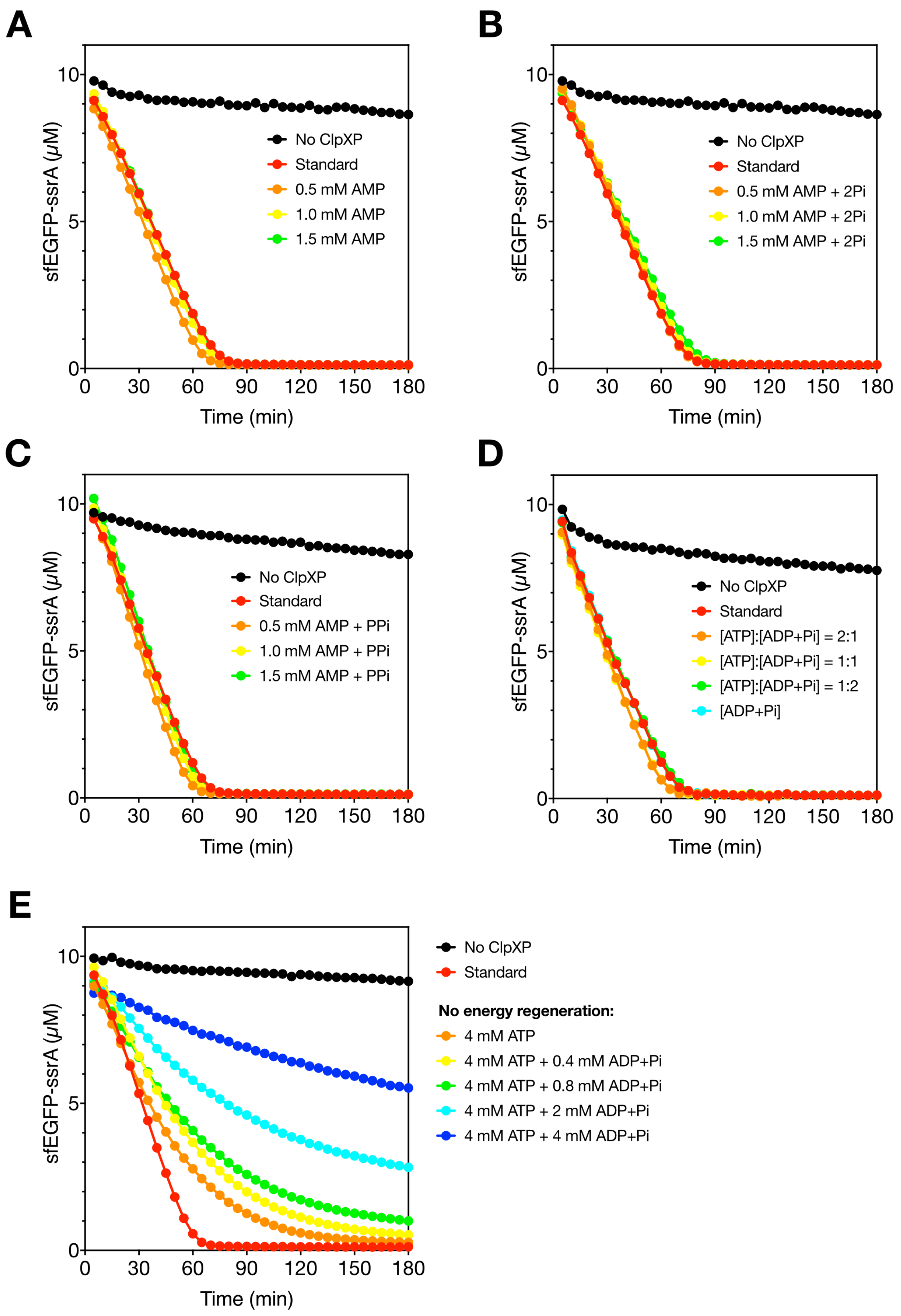


Supplementary Figure S6
